# Dynamic Quantitative Trait Loci Mapping for Plant Height in Recombinant Inbred Line Population of Upland Cotton

**DOI:** 10.3389/fpls.2022.914140

**Published:** 2022-06-09

**Authors:** Jing Wu, Lili Mao, Jincai Tao, Xiuxiu Wang, Haijun Zhang, Ming Xin, Yongqi Shang, Yanan Zhang, Guihua Zhang, Zhongting Zhao, Yiming Wang, Mingshuo Cui, Liming Wei, Xianliang Song, Xuezhen Sun

**Affiliations:** ^1^State Key Laboratory of Crop Biology, Agronomy College, Shandong Agricultural University, Taian, China; ^2^State Key Laboratory of Crop Stress Biology for Arid Areas, College of Agronomy, Northwest Agriculture and Forestry University, Xianyang, China; ^3^Heze Academy of Agricultural Sciences, Heze, China

**Keywords:** *Gossypium hirsutum*, recombinant inbred line, plant height, genetic map, quantitative trait loci, candidate gene

## Abstract

Plant height (PH) is a key plant architecture trait for improving the biological productivity of cotton. Ideal PH of cotton is conducive to lodging resistance and mechanized harvesting. To detect quantitative trait loci (QTL) and candidate genes of PH in cotton, a genetic map was constructed with a recombinant inbred line (RIL) population of upland cotton. PH phenotype data under nine environments and three best linear unbiased predictions (BLUPs) were used for QTL analyses. Based on restriction-site-associated DNA sequence (RAD-seq), the genetic map contained 5,850 single-nucleotide polymorphism (SNP) markers, covering 2,747.12 cM with an average genetic distance of 0.47 cM. Thirty-seven unconditional QTL explaining 1.03–12.50% of phenotypic variance, including four major QTL and seven stable QTL, were identified. Twenty-eight conditional QTL explaining 3.27–28.87% of phenotypic variance, including 1 major QTL, were identified. Importantly, five QTL, including 4 stable QTL, were both unconditional and conditional QTL. Among the 60 PH QTL (including 39 newly identified), none of them were involved in the whole period of PH growth, indicating that QTL related to cotton PH development have dynamic expression characteristics. Based on the functional annotation of *Arabidopsis* homologous genes and transcriptome data of upland cotton TM-1, 14 candidate genes were predicted within 10 QTL. Our research provides valuable information for understanding the genetic mechanism of PH development, which also increases the economic production of cotton.

## Introduction

As the most important natural textile fiber, cotton (*Gossypium* spp.) supplies about 35% of global fiber consumption, which is also used to make feed and oilseeds, and upland cotton (*Gossypium hirsutum* L.) accounts for more than 90% of global cotton production (Chen et al., 2007; Wang et al., 2015; Zhang et al., 2015; Ma et al., 2018b). Cotton production is a part of the cash crop industry (Li, 2021). With the change of the cotton industry trade situation and the promotion of mechanization, it is very important to cultivate high-yield and good-quality cotton varieties. Plant architecture breeding can effectively increase cotton yield and improve the fiber quality, thereby affecting the economic production of cotton (Li et al., 1998, 2015; Song and Zhang, 2009; Shang et al., 2015b; Fu et al., 2019). Plant architecture refers to the three-dimensional structure of the aerial part of the plant, which is a comprehensive agronomic trait, including many factors such as PH, fruit branch number, angle between stem and fruit branch, morphological characters and distribution of boll and leaf, and so forth (Yang et al., 1996; Wang and Li, 2006; Wu et al., 2021). It is worth noting that the Green Revolution that emerged in the 1960s changed the plant architecture by transferring dwarf genes into crops, resulting in a significant increase in yields (Khush, 2001). For example, reducing PH enhanced resistance and significantly increased yield of wheat and rice (Peng et al., 1999; Sasaki et al., 2002). Besides, using the compact and medium dwarf corn varieties increased planting density and significantly improved the production (Peiffer et al., 2014).

Plant height (PH) is a main factor affecting the plant architecture (Jiao et al., 2010; Zhang et al., 2019). In addition, the appropriate PH of cotton (80–90 cm) is also beneficial for lodging resistance and mechanical harvesting (Chen, 2013; Pei et al., 2021). Therefore, a study on PH of cotton will be helpful for cotton plant architecture breeding. However, PH is a quantitative trait controlled by multiple genes and affected by the environment easily (Cui et al., 2011; Wurschum et al., 2015; Liu et al., 2018). With the rapid development of molecular marker technology, the molecular genetic mechanism of quantitative trait can be studied by the linkage analysis and association analysis. QTL-seq and QTL mapping techniques were used to identify the main PH QTL on the A10 chromosome in two F_2_ populations of *Brassica napus* (Dong et al., 2021). In maize, a major QTL controlling PH was mapped by three different methods, including genome wide association study (GWAS), and was located in a 600 kb region (Pan et al., 2017). In wheat, seven stable PH QTL were detected on six chromosomes, among which *QPht.cau-3D1* explaining 28.36–38.08% of phenotypic variance was stable QTL (Chen et al., 2020). In rice, a PH QTL *qPH7-2* explaining 36.77–56.82% of phenotypic variance was detected in an RIL population under three environments (Lei et al., 2018). The molecular genetic mechanism of cotton PH is mainly studied by the linkage analysis. Fourteen PH QTL were identified on 11 chromosomes of an upland cotton F_2_ population, with 9.64% of the contribution rate of *qPH-D12-1* (Jia et al., 2021). A stable QTL *qPH-Dt1-1*, as well as a gene *GhPIN3* regulating cotton PH, was identified in an interspecific *G. hirsutum* × *G. barbadense* BIL population using the SLAF-seq technology (Ma et al., 2019). Twenty-seven PH QTL were mapped on 18 chromosomes using an RIL population of upland cotton, with 3.81–8.54% of phenotypic variance (Liu et al., 2018). In addition, naturally dwarfing cotton mutants are very important for identifying PH genes of cotton. A gene *EXTR-DWARF* related to dwarfing was screened from a natural dwarf mutant of cotton (Ji, 2018).

Cotton PH QTL previously reported were mainly mapped on phenotypic PH data of mature plants, which largely ignored the dynamic development process of PH. The net effect of QTL expression in the time period from t-1 to t may be evaluated based on the time-related genetic model of quantitative traits (Zhu, 1995). Forty-one unconditional QTL and 23 conditional QTL were detected using an intraspecific recombinant inbred line population of upland cotton, and some QTL could not be detected at the later stage of maturity (Shang et al., 2015b). Recently, thirty-two unconditional QTL and 24 conditional QTL for PH were detected using an intraspecific testcross population of upland cotton (Ma et al., 2018a). These results indicated that the expression of PH QTL during plant growth has temporal characteristics.

At present, there are relatively few studies on the dynamic QTL mapping of cotton PH. In this study, unconditional QTL and conditional QTL for PH in different environments and developmental stages were detected, and candidate genes affecting PH development in cotton were predicted. Our results shed substantial light on the genetic basis of cotton PH, will lay a foundation for further functional verification of candidate genes, and enable molecular selection toward ideal plant architecture.

## Materials and Methods

### Plant Materials

An F_2:7_ RIL population, containing 201 lines coming from an intraspecific upland cotton cross of WCT-4 (female) × NK-4 (male), was constructed using a single seed descent method. The WCT-4 has short PH and super fiber quality, and the NK-4 is tall with average fiber quality. Both parents were developed by the Cotton Breeding & Cultivation team of Shandong Agricultural University. The WCT-4 is a strain from the cross of Shannong269//(Bersiruo/Xinhai2hao) F_4_. The NK-4 is a strain from the cross Shannong6/Shiyuan321//shannong6. The RIL population with two parents were grown in nine environments across two locations and 5 years in Shandong Province, China, including Heze from 2017 to 2021 (17HZ, 18HZ, 19HZ, 20HZ, 21HZ), and Dezhou from 2018 to 2021 (18DZ, 19DZ, 20DZ, and 21DZ). The materials planting followed a randomized complete block design with two replications at each location under all environments. Each replicate had one row of 5 m. The row width and average plant space were 0.9 and 0.23 m in HZ in all 5 years and were 0.8 and 0.25 m in DZ in all 4 years. Planting dates for all environments were April 24–28. Local cultivation management methods were used in each location.

### Phenotypic Measurements and Analysis

Plant height refers to the length from the cotyledonary node to the main stem growth point. Dynamic PH was investigated four times during cotton growth, with an interval of about 10 days, in all environments except for in 17HZ, 19DZ, 21HZ, and 21DZ, of which only three times array were carried out. The investigation time points were t1: June 25th, t2: July 5th, t3: July 15 in 19DZ, 21HZ, and 21DZ, and t2: July 5th, t3: July 15th, t4: July 25th in 17HZ, and t1: June 25, t2: July 5, t3: July 15, t4: July 25 in other environments. △t1-2 referred to the net increase of PH between t1 and t2, △t2-3 was the net increase of PH from t2 to t3, and △t3-4 was the net increase of PH from t3 to t4.

The descriptive data of RIL population PH traits were sorted out and calculated by IBM SPSS Statistics 25. Trait variance and generalized heritability (*H*^2^) were calculated with the QTL IciMapping 4.2 (Meng et al., 2015; Ma et al., 2019). Generalized heritability above 40%, between 20 and 40%, and less than 20% were regarded as high, medium, and low heritability, respectively (Wang et al., 2018).

### Genetic Map Construction

Sample DNA was extracted using a plant genomic DNA extraction kit. SNP markers were developed through RAD-seq at ROI-BIO Co., Ltd. The parental NK-4 and WCT-4 obtained 41.09 and 38.94 Gb clean reads, respectively. For each RIL line, 2.22–15.68 Gb clean reads were obtained. The genetic map was constructed with an RIL population (F_2:8_ generation), and the SNP markers were filtered according to the sequencing results. First, the parental heterozygous markers, no polymorphic markers between the two parents, the bi-allelic markers, and the markers with a deletion rate of greater than 50% were eliminated. Then, the linkage group was constructed on the LOD value, and then the markers that were too far to be used to construct the genetic map were deleted. The genetic map was constructed with the Kosambi mapping function in JoinMap 4.0 (Ooijen, 2006) and created by MapChart 2.2 (Voorrips, 2002). The TM-1 genome sequence of Zhejiang University (Hu et al., 2019) was downloaded from the CottonFGD.^1^ TBtools was used to extract the genome chromosome length information and draw the collinearity relationship diagram (Chen et al., 2018). IBM SPSS Statistics 25 was used to calculate the Spearman coefficient.

### Quantitative Trait Loci Analysis

The PH data of the RIL population under nine environments and their BLUPs (named as BLUP3) across all the tests were used for QTL analysis. Two other BLUPs, namely, BLUP1 for HZ experimental site and BLUP2 for DZ site, were also estimated and used for QTL analysis to detect specific QTL of the experimental site. The lme4 package in the R software was used to estimate the BLUPs (de Los Campos et al., 2013; Huang et al., 2017). Unconditional QTL was the cumulative effect from the initial time to t (Liu, 2011), which was detected by t1, t2, t3, and t4, respectively. The net genetic effect of PH increase from t-1 to t was revealed by the conditional genetic analysis (Zhu, 1995). The growth of PH in the past adjacent time period (△t1-2, △t2-3, and △t3-4) were used to identify conditional QTL. Consistent QTL were determined by integrating unconditional QTL and conditional QTL. QTL were identified based on the PH value of nine environments and three BLUPs by the ICIM-ADD method in QTL IciMapping 4.2 (Meng et al., 2015), with 1 cM of the parameter step value, 0.05 value of P of type I error, and 0.01 value of the PIN; meanwhile, the threshold was calculated in 1,000 permutations for each trait. The QTL confidence interval (95%) was set as the mapping distance interval corresponding to reduce 1 LOD on either side of the peak (Yu et al., 2012; Liu et al., 2019). When the QTL confidence intervals partially or completely overlap, it was regarded as the same QTL. QTL detected in two or more tests or environments with LOD value greater than 2.5 and phenotypic variation explanation rate greater than 10% were defined as major QTL. It was regarded as stable QTL, which was detected in at least three environments (Sun et al., 2012; Fan et al., 2015; Liu et al., 2018). The name of QTL starts with *q*, followed by the trait name, chromosome, and QTL number (McCouch et al., 1997), and the names of unconditional QTL and conditional QTL were respectively prefixed with *U* and *C*. If a conditional QTL shared confidence interval with an unconditional QTL, these two QTL were renamed as one QTL without prefixed both *U* and *C* in the name. The QTL were displayed on the genetic map by MapChart 2.2 (Voorrips, 2002). GraphPad Prism 9 was used to do boxplot of QTL with phenotypic data of BLUP3. The positive additive effect of QTL indicated that the positive allele was derived from the female parent WCT-4; otherwise, the positive allele was derived from the male parent NK-4.

### Candidate Gene Analysis

To annotate the gene function, we used the COTTONOMICS database^2^ to extract candidate genes from the confidence intervals of QTL with large effect. TBtools and omicstudio^3^ were used to do the Gene Ontology (GO) enrichment analysis on genes, while novomagic^4^ was utilized to perform the Kyoto Encyclopedia of Genes and Genomes (KEGG) analysis on genes. To uncover the general expression patterns of candidate genes, the transcriptome data of TM-1 roots, stems, and leaves were used as a reference (Hu et al., 2019). TBtools was used to create the genes expression heatmap (Chen et al., 2018).

## Results

### Plant Height Variation of Parents and the Recombinant Inbred Line Population

The PH of male parent NK-4 was higher than that of female parent WCT-4 at all the time points (i.e., t1, t2, t3, and t4) and most time periods (18/23) in the nine environments. The PH of the RIL population showed a two-way transgressive segregation in all time points under all environments except for the t1 in 17HZ with a one-way transgressive segregation (Table 1). The values of skewness and kurtosis in each environment were close to 0, and the PH of the RIL population showed a normal distribution. Under the nine environments, the PH of the parents and RIL population increased rapidly in the △t1-2 and △t2-3, with an average growth of 19.48 and 13.90 cm, and slowed down in the △t3-4, with an average increase of 5.97 cm (Table 1). The results showed that the PH of parents and RIL population had dynamic developmental characteristics. The analysis of ANOVA on the RIL population showed that the environment, genotype, and genotype × environment had significant variations on PH at t1, t2, t3, and t4 (*P* < 0.01) (Supplementary Table 1). Furthermore, the generalized heritability estimates (*H*^2^) were 78.19, 83.62, 85.57, and 84.78% at t1, t2, t3, and t4, respectively, suggesting that PH was highly heritable in this RIL population (Supplementary Table 1).

**TABLE 1:** Plant height performance of recombinant inbred lines (RILs) and their parents.

Environment	Stage	Parents			RIL population						
		NK-4	WCT-4	P_*N*_-P_*W*_	Minimum	Maximum	Mean	SD	CV/%	Skewness	Kurtosis
17HZ	t2	77.03	55.33	21.70	55.60	86.10	70.92	6.10	8.60	–0.02	–0.36
	t3	91.76	67.66	24.10	66.70	98.80	83.97	6.68	7.96	0.04	–0.33
	t4	98.52	77.69	20.83	73.30	104.00	89.91	6.41	7.13	–0.25	–0.10
18HZ	t1	78.40	71.90	6.50	51.30	87.30	68.79	6.82	9.92	0.06	–0.16
	t2	103.67	86.35	17.32	73.40	111.60	90.81	7.24	7.97	0.04	–0.23
	t3	110.47	92.89	17.58	78.20	115.90	96.93	6.76	6.98	–0.09	–0.04
	t4	113.10	95.84	17.26	84.90	124.60	102.18	6.73	6.59	0.12	0.20
18DZ	t1	61.83	42.08	19.75	30.50	67.22	50.54	7.12	14.09	–0.13	0.10
	t2	81.75	54.08	27.67	45.60	86.20	66.54	8.01	12.03	0.02	–0.27
	t3	92.88	65.17	27.71	55.30	99.00	77.21	8.39	10.86	0.05	–0.25
	t4	96.39	69.79	26.61	62.80	106.60	84.32	9.23	10.95	0.21	–0.39
19HZ	t1	73.47	63.43	10.03	48.50	81.70	66.41	6.62	9.97	–0.09	–0.49
	t2	90.83	77.32	13.51	54.29	99.50	82.57	8.03	9.73	–0.27	0.29
	t3	95.47	80.53	14.93	58.43	107.50	88.10	7.96	9.04	–0.31	0.39
	t4	97.33	82.20	15.13	62.43	108.13	90.47	7.84	8.66	–0.27	0.28
19DZ	t1	42.50	39.74	2.76	32.33	75.50	48.67	8.41	17.28	0.40	0.21
	t2	62.26	57.60	4.66	44.40	103.40	68.09	10.68	15.68	0.22	0.53
	t3	88.46	75.28	13.18	62.00	124.60	89.66	11.55	12.88	–0.06	–0.05
20HZ	t1	63.23	50.87	12.37	37.50	71.90	52.25	6.21	11.89	0.24	0.11
	t2	82.97	66.07	16.90	51.80	88.60	69.42	7.03	10.13	0.03	0.10
	t3	98.53	80.03	18.50	64.40	102.10	84.13	7.50	8.91	–0.20	–0.03
	t4	108.17	88.19	19.97	71.70	110.60	93.31	7.83	8.39	–0.50	0.01
20DZ	t1	54.23	45.80	8.43	35.50	61.60	47.54	5.40	11.36	0.09	–0.45
	t2	78.19	68.17	10.01	49.70	90.30	70.02	7.80	11.14	0.04	–0.35
	t3	96.70	75.67	21.03	52.20	110.60	81.87	9.20	11.24	0.02	0.43
	t4	102.57	79.97	22.60	61.70	114.10	87.87	9.35	10.64	0.04	–0.08
21HZ	t1	53.90	50.00	3.90	35.10	63.60	50.04	5.91	11.80	–0.02	–0.33
	t2	75.77	74.50	1.27	50.40	93.60	72.42	7.98	11.02	0.12	0.07
	t3	97.47	91.30	6.17	64.00	115.40	91.80	9.60	10.46	–0.26	0.02
21DZ	t1	62.27	51.50	10.77	21.40	71.20	52.66	7.84	14.89	–0.13	0.40
	t2	86.17	70.60	15.57	34.00	95.90	73.23	10.02	13.69	–0.18	0.17
	t3	105.67	90.67	15.00	46.40	121.80	95.58	10.76	11.25	–0.39	1.24
17HZ	△t2–3	14.73	12.32	2.40	5.20	23.90	13.05	3.07	23.52	0.25	0.72
	△t3-4	6.76	10.03	–3.28	0.20	14.70	5.94	2.73	45.95	0.27	0.00
18HZ	△t1-2	25.27	14.45	10.82	11.50	30.00	22.02	3.56	16.17	–0.14	0.07
	△t2-3	6.80	6.54	0.26	0.10	14.29	6.13	2.82	46.06	0.21	–0.26
	△t3-4	2.63	2.95	–0.32	0.10	16.70	5.24	2.44	46.45	0.60	1.95
18DZ	△t1-2	19.92	12.00	7.92	5.90	33.20	15.59	4.33	27.75	1.07	2.75
	△t2-3	11.13	11.09	0.04	1.60	24.90	10.67	4.10	38.43	0.19	0.08
	△t3-4	3.51	4.62	–1.11	0.10	18.30	7.11	3.49	49.15	0.26	0.07
19HZ	△t1-2	17.37	13.89	3.48	4.12	26.40	16.16	3.43	21.23	–0.13	0.86
	△t2-3	4.63	3.21	1.42	2.00	11.10	5.53	1.79	32.30	0.54	0.14
	△t3-4	1.87	1.67	0.20	–0.02	7.90	2.37	1.73	72.69	0.78	–0.07
19DZ	△t1-2	19.76	17.86	1.90	10.10	36.10	19.42	4.38	22.53	0.60	0.40
	△t2-3	26.20	17.68	8.52	9.41	33.80	21.57	5.00	23.20	–0.11	–0.29
20HZ	△t1-2	19.73	15.20	4.53	8.60	24.60	17.17	3.27	19.04	–0.04	–0.56
	△t2-3	15.57	13.97	1.60	8.10	23.80	14.71	3.03	20.60	0.32	0.20
	△t3-4	9.63	8.16	1.48	0.80	17.40	9.18	3.03	33.02	0.08	0.00
20DZ	△t1-2	23.95	22.37	1.58	10.00	29.80	22.48	4.03	17.91	–0.62	0.80
	△t2-3	18.51	7.49	11.02	2.50	23.38	11.85	4.12	34.77	0.58	0.08
	△t3-4	5.87	4.30	1.57	0.27	16.67	5.99	3.72	62.01	0.62	–0.24
21HZ	△t1-2	21.87	24.50	–2.63	12.70	32.60	22.39	3.72	16.63	0.19	–0.14
	△t2-3	21.70	16.80	4.90	11.20	29.30	19.37	3.76	19.42	0.07	–0.56
21DZ	△t1-2	23.90	19.10	4.80	9.10	30.40	20.57	3.94	19.16	–0.12	0.22
	△t2-3	19.50	20.07	–0.57	1.11	29.30	22.21	4.23	19.05	–1.05	3.15

*HZ, Heze, Shandong (2017, 2018, 2019, 2020, 2021); DZ, Dezhou, Shandong (2018, 2019, 2020, 2021).*

### Genetic Map Construction

**FIGURE 1:**
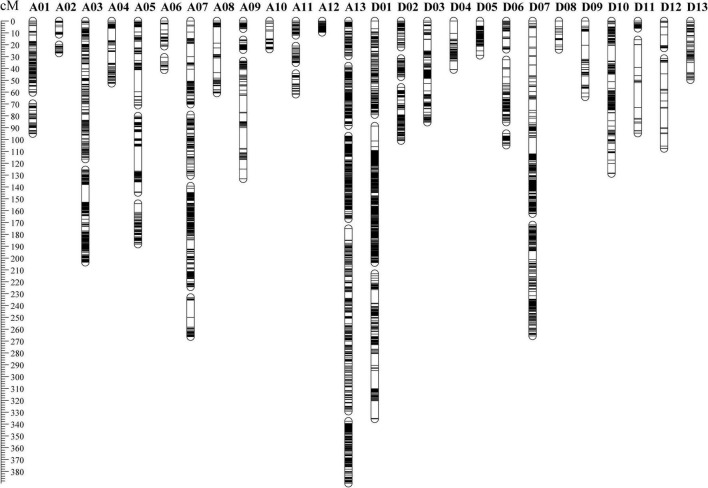
The distribution of polymorphic markers in the genetic map constructed by the recombinant inbred line population.

By comparing the RAD-seq sequencing results with the reference genome of TM-1, a total of 555,699 SNP markers were obtained. According to the genotyping criteria, SNP marker filtering was performed. First, 87,613 SNP markers remained after filtering out SNP markers that were heterozygous and non-polymorphic between parents, and then, 87,596 SNP markers remained after filtering out SNP markers of diallelic sites. Finally, after filtering out SNP markers with a deletion rate greater than 50%, 10,054 SNP markers were used for genetic map construction (Supplementary Table 2). A high-density genetic map of 5,850 SNP markers was consequently constructed. The genetic map included 52 linkage groups (LGs), and A13 contained the most SNP markers (1,067), followed by D01 (730) (Supplementary Table 3). The total length of the genetic map was 2,747.12 cM with an average genetic distance of 0.47 cM between adjacent markers (Figure 1 and Supplementary Table 3). The longest LG was D07-1, which spanned 162.45 cM and contained 290 SNP markers with an average of 0.56 cM. The shortest LG was D11-1, which spanned 6.21 cM and contained 26 SNP markers with an average of 0.24 cM.

**FIGURE 2:**
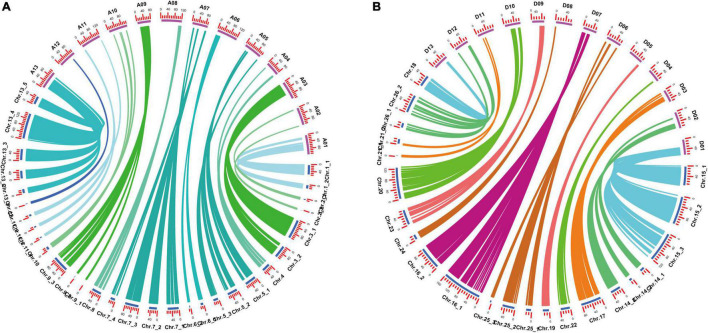
Collinearity analysis of genetic map and physical map. **(A)** A subgenomic chromosome collinearity. **(B)** D subgenomic chromosome collinearity.

Among the 52 LGs, 24 LGs had Spearman coefficient greater than 0.80, and 26 LGs had Spearman coefficient greater than 0.70 (Supplementary Table 3). Spearman coefficient of each LG was found to have an average value of 0.80, indicating a high degree of collinearity between the genetic map and the physical map. It indicated that the order of most markers in the genetic map was consistent with the order in the physical map, according to the collinearity analysis based on the physical location on the TM-1 reference genome and the position in the linkage group of the genetic map (Figure 2 and Supplementary Table 3).

### Unconditional Quantitative Trait Loci Mapping

**FIGURE 3:**
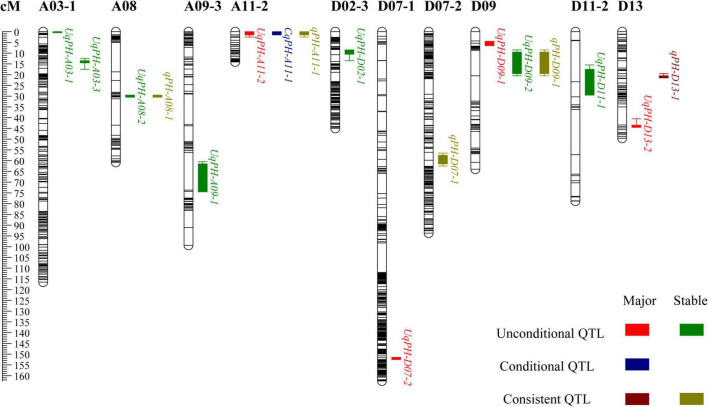
The chromosome-wise distribution of plant height quantitative trait loci (QTL).

In total, 12 sets of phenotypic data of PH, including phenotypic data from nine testing environments and three BLUPs (i.e., BLUP1, BLUP2, and BLUP3) were used to detect QTL. At four time points (i.e., t1, t2, t3, and t4), a total of 37 unconditional QTL (20 on the A subgenome and 17 on the D subgenome) were identified on 14 chromosomes, and each QTL explained 1.03–12.50% of phenotypic variance with LOD value varying from 3.33 to 27.26 (Supplementary Table 4). Among the 37 QTL, WCT-4 contributed positive additive alleles for 19 QTL, while NK-4 provided positive additive alleles for the rest 18 QTL. Importantly, 7 QTL (i.e., *UqPH-A03-1*, *UqPH-A03-3*, *UqPH-A08-2*, *UqPH-A09-1*, *UqPH-D02-1*, *UqPH-D09-2*, and *UqPH-D11-1*) were simultaneously detected at least in 3 tests and considered to be stable QTL (Table 2 and Figure 3). Moreover, other 30 QTL were identified in one or two tests, including four major QTL (i.e., *UqPH-A11-2*, *UqPH-D07-2*, *UqPH-D09-1*, and *UqPH-D13-2*). There were 16, 24, 19, and 14 QTL detected at t1, t2, t3, and t4, respectively, suggesting that there were different QTL at different stages of PH development.

**TABLE 2:** Major and stable quantitative trait loci (QTL) detected for plant height in different environments.

QTL name	Environment	Stage	Position (cM)	LeftMarker	RightMarker	LOD	PVE (%)	Additive
*UqPH-A03-1*	19DZ	t3	0	A03-1154016	A03-1152903	7.44	3.03	–4.69
	20DZ	t4	0	A03-1154016	A03-1152903	5.44	1.85	–2.79
	BLUP1	t1	0	A03-1154016	A03-1152903	11.51	5.40	–1.03
	BLUP3	t1	0	A03-1154016	A03-1152903	14.11	6.82	–1.33
		t2	0	A03-1154016	A03-1152903	18.88	7.87	–2.34
*UqPH-A03-3*	19HZ	t1	14	A03-1312663	A03-1163998	3.74	5.85	1.77
		t3	14	A03-1312663	A03-1163998	19.48	7.37	5.28
	19DZ	t3	16	A03-1310900	A03-1310888	14.61	6.43	6.98
	20DZ	t4	13	A03-1471008	A03-1312663	11.38	4.14	4.23
	BLUP1	t1	16	A03-1310900	A03-1310888	19.27	9.95	1.42
	BLUP3	t1	16	A03-1310900	A03-1310888	19.46	10.04	1.64
		t2	17	A03-1310900	A03-1310888	23.19	10.19	2.72
*UqPH-A08-2*	17HZ	t3	30	A08-118502121	A08-118512814	3.71	4.84	–1.72
	19HZ	t1	30	A08-118502121	A08-118512814	5.45	8.82	–2.13
	21HZ	t1	30	A08-118502121	A08-118512814	3.42	2.02	–1.33
		t2	30	A08-118502121	A08-118512814	4.58	1.55	–2.12
		t3	30	A08-118502121	A08-118512814	5.39	1.39	–2.55
	BLUP1	t1	30	A08-118502121	A08-118512814	5.37	2.36	–0.67
		t2	30	A08-118502121	A08-118512814	8.58	4.13	–1.19
		t3	30	A08-118502121	A08-118512814	8.20	7.76	–1.09
		t4	30	A08-118502121	A08-118512814	6.28	6.39	–0.90
	BLUP3	t1	30	A08-118502121	A08-118512814	5.60	2.45	–0.79
		t2	30	A08-118502121	A08-118512814	3.55	1.23	–0.92
*UqPH-A09-1*	20DZ	t3	70	A09-61223718	A09-62602002	3.65	5.53	2.70
		t4	70	A09-61223718	A09-62602002	3.83	1.62	2.60
	BLUP2	t3	70	A09-61223718	A09-62602002	3.87	1.49	0.90
*UqPH-A11-2*	17HZ	t3	0	A11-5274523	A11-5136456	3.90	5.09	–1.79
	20DZ	t4	2	A11-5136456	A11-5095875	20.81	8.49	–6.01
*UqPH-D02-1*	18HZ	t3	11	D02-60076418	D02-60261295	3.62	4.11	–1.74
	21HZ	t2	9	D02-58357588	D02-60076418	10.68	3.98	-3.41
		t3	9	D02-58357588	D02-60076418	6.04	1.62	–2.76
*UqPH-D07-2*	BLUP2	t2	152	D07-14496431	D07-14713797	12.86	4.05	0.53
		t3	152	D07-14496431	D07-14713797	19.90	7.66	2.08
*UqPH-D09-1*	18DZ	t2	6	D09-36097451	D09-35948718	10.24	5.37	3.39
	BLUP2	t4	5	D09-36092845	D09-36097451	9.64	4.92	1.15
*UqPH-D09-2*	18DZ	t2	9	D09-35991824	D09-38656333	20.68	12.50	–5.17
		t3	14	D09-35991824	D09-38656333	3.47	6.48	–2.52
	BLUP2	t4	10	D09-35991824	D09-38656333	19.01	11.34	–1.75
	BLUP3	t4	15	D09-35991824	D09-38656333	4.26	6.25	–1.35
*UqPH-D11-1*	17HZ	t2	25	D11-54584560	D11-54120704	5.54	4.00	1.95
		t3	25	D11-54584560	D11-54120704	5.05	7.45	2.14
	BLUP3	t4	23	D11-25798969	D11-54584560	3.45	3.87	1.07
*UqPH-D13-2*	21HZ	t2	43	D13-50103125	D13-53665080	13.05	5.01	–3.81
		t3	44	D13-53665080	D13-53664776	19.45	5.97	–5.28
*CqPH-A11-1*	17HZ	t2-3	0	A11-5274523	A11-5136456	7.67	4.91	–1.08
	BLUP3	t2-3	1	A11-5274523	A11-5136456	4.96	5.99	–0.15

*Uq and Cq refer to unconditional and conditional QTL, respectively; HZ refers to Heze, Shandong (2017, 2018, 2019, 2020, 2021); DZ refers to Dezhou, Shandong (2018, 2019, 2020, 2021); BLUP1 refers to the best linear unbiased predicted value calculated in the 5 Heze environments; BLUP2 refers to the best linear unbiased predicted value calculated in the 4 Dezhou environments; BLUP3 refers to the best linear unbiased predicted value calculated in the nine environments; and PVE and additive means the explanation of phenotypic variation and additive effect, respectively.*

### Conditional Quantitative Trait Loci Mapping

In 3 time periods (i.e., △t1-2, △t2-3, and △t3-4) across the 12 sets of phenotypic data of PH, a total of 28 conditional QTL were identified on 17 chromosomes, which explained 3.27–28.87% of phenotypic variance with LOD value ranging from 3.18 to 28.78 (Supplementary Table 5). Among the 28 QTL, 13 and 15 QTL were detected on the A subgenome and the D subgenome, respectively. The alleles from WCT-4 for 15 QTL had positive additive effects on PH, while alleles from NK-4 for other 13 QTL had positive additive effects on PH. Furthermore, *CqPH-A11-1* was identified in 2 tests as the major QTL explaining 4.91–5.99% of phenotypic variance with an LOD value ranging from 4.96 to 7.67 (Table 2 and Figure 3). Seven QTL and 23 QTL were detected in △t1-2 and △t2-3, respectively, and no QTL was detected in △t3-4, indicating that conditional QTL were more positively expressed in △t2-3.

### Consistent Quantitative Trait Loci Mapping

By compared 37 unconditional QTL with 28 conditional QTL, 5 QTL (i.e., *qPH-A08-1*, *qPH-A11-1*, *qPH-D07-1*, *qPH-D09-1*, and *qPH-D13-1*) shared overlapping or common confidence intervals and were regarded as consistent QTL (Table 3 and Figure 3). In summary, a total of 60 QTL (31 on the A subgenome and 29 on the D subgenome) were identified across the 12 sets of phenotypic data of PH (Supplementary Table 6). In addition, no QTL could be detected at all stages in the same environment, which accorded with the characteristics of PH dynamic development.

**TABLE 3:** Consistent quantitative trait loci (QTL) of plant height detected in different environments.

Meta-QTL name	Environment	Stage	Position (cM)	LeftMarker	RightMarker	LOD	PVE (%)	Additive
*qPH-A08-1*	17HZ	t3	30	A08-118502121	A08-118512814	3.71	4.84	–1.72
	19HZ	t1	30	A08-118502121	A08-118512814	5.45	8.82	–2.13
	21HZ	t1	30	A08-118502121	A08-118512814	3.42	2.02	–1.33
		t2	30	A08-118502121	A08-118512814	4.58	1.55	–2.12
		△t2-3	30	A08-118502121	A08-118512814	4.77	4.70	–1.12
		t3	30	A08-118502121	A08-118512814	5.39	1.39	–2.55
	BLUP1	t1	30	A08-118502121	A08-118512814	5.37	2.36	–0.67
		t2	30	A08-118502121	A08-118512814	8.58	4.13	–1.19
		t3	30	A08-118502121	A08-118512814	8.20	7.76	–1.09
		t4	30	A08-118502121	A08-118512814	6.28	6.39	–0.90
	BLUP3	t1	30	A08-118502121	A08-118512814	5.60	2.45	–0.79
		t2	30	A08-118502121	A08-118512814	3.55	1.23	–0.92
*qPH-A11-1*	17HZ	△t2-3	0	A11-5274523	A11-5136456	7.67	4.91	–1.08
		t3	0	A11-5274523	A11-5136456	3.90	5.09	–1.79
	20DZ	t4	2	A11-5136456	A11-5095875	20.81	8.49	–6.01
	BLUP3	△t2-3	1	A11-5274523	A11-5136456	4.96	5.99	–0.15
*qPH-D07-1*	19DZ	t2	60	D07-33680166	D07-32594728	5.57	1.03	–3.59
	20HZ	△t1-2	62	D07-33680166	D07-32594728	25.95	4.46	2.38
	BLUP1	t2	60	D07-33680166	D07-32594728	14.69	7.68	–1.63
*qPH-D09-1*	18DZ	△t1-2	12	D09-35991824	D09-38656333	5.05	4.76	–1.54
		t2	9	D09-35991824	D09-38656333	20.68	12.50	–5.17
		t3	14	D09-35991824	D09-38656333	3.47	6.48	–2.52
	BLUP2	t4	10	D09-35991824	D09-38656333	19.01	11.34	–1.75
	BLUP3	t4	15	D09-35991824	D09-38656333	4.26	6.25	-1.35
*qPH-D13-1*	17HZ	△t2-3	20	D13-49336750	D13-49673501	9.04	5.96	–1.19
	21HZ	t1	21	D13-49673501	D13-50097370	5.24	3.17	1.68

*Refer to Table 2 for notes.*

Stable and reliable QTL/alleles across different periods are of great value in marker assisted breeding. For the five consistent QTL, *qPH-A08-1*, *qPH-A11-1*, *qPH-D07-1*, *qPH-D09-1*, and *qPH-D13-1*, markers A08-118512814, A11-5136456, D07-33680166, D09-35991824, and D13-49673501 were the nearest linked SNP markers, respectively (Table 3 and Supplementary Figure 1). For SNP marker A08-118512814 for *qPH-A08-1*, RIL lines with SNP allele genotype (bb) from NK-4 had significantly higher PH than RIL lines with SNP allele genotype (aa) from WCT-4 in 3 of 5 periods (*P* < 0.05) (Supplementary Figure 1a). For SNP marker A11-5136456 for *qPH-A11-1*, the average PH of RIL lines with SNP allele genotype (bb) from NK-4 was significantly higher than that of RIL lines with SNP allele genotype (aa) from WCT-4 in 2 of 3 periods (*P* < 0.05) (Supplementary Figure 1b). For SNP marker D09-35991824 for *qPH-D09-1*, the average PH of RIL lines with SNP allele genotype (bb) from NK-4 was significantly higher than that of RIL lines with SNP allele genotype (aa) from WCT-4 in 1 of 4 periods (*P* < 0.05) (Supplementary Figure 1d). However, for D07-33680166 (*qPH-D07-1*) and D13-49673501 (*qPH-D13-1*), the difference between the average PH of RIL lines with the SNP allele genotype (aa) from WCT-4 and with SNP allele genotype (bb) from NK-4 was not significant (Supplementary Figures 1c,e). These results indicated that *qPH-A08-1*, *qPH-A11-1*, and *qPH-D09-1* had relatively reliable effects on PH.

### Gene Enrichment

**FIGURE 4:**
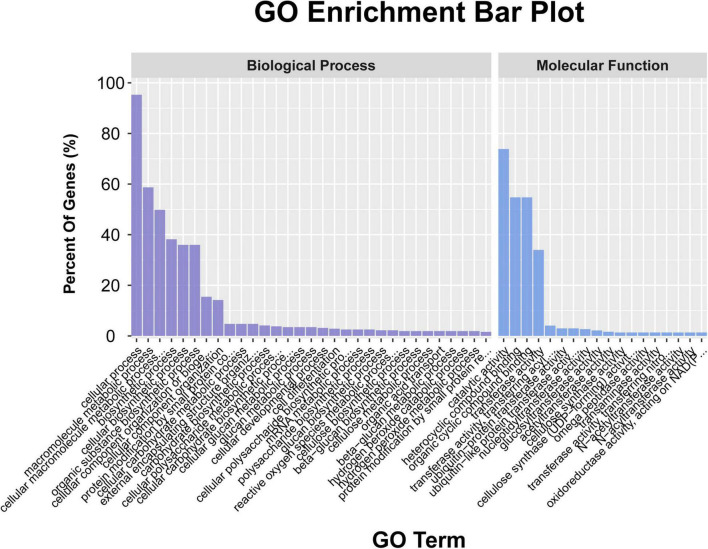
The annotation of the candidate genes in the ten quantitative trait loci (QTL) through gene ontology (GO) analysis.

A total of 948 genes were identified within the physical interval of the 5 stable unconditional QTL (i.e., *UqPH-A03-1*, *UqPH-A03-3*, *UqPH-A09-1*, *UqPH-D02-1*, and *UqPH-D11-1*) and five consistent QTL (i.e., *qPH-A08-1*, *qPH-A11-1*, *qPH-D07-1*, *qPH-D09-1*, and *qPH-D13-1*) (Supplementary Tables 6, 7). The GO enrichment results (*P* < 0.05) showed that 317 genes were enriched in the biological process category, with 302 genes participated in cellular processes, 186 genes participated in macromolecule metabolic processes, and 158 genes participated in cellular macromolecule metabolic processes, respectively. A total of 371 genes were enriched in the molecular function category, with 274 genes were associated with catalytic activity, 203 genes were associated with heterocyclic compound binding and organic cyclic compound binding respectively, and 126 genes were associated with transferase activity (Figure 4 and Supplementary Table 8). The KEGG enrichment results (*P* < 0.05) showed that the most likely pathway was SNARE interactions in vesicular transport (enriched in six genes) (Supplementary Table 9).

### Plant Height Candidate Genes Prediction

To predict candidate genes associated with cotton PH, homologous genes functional annotation of *Arabidopsis* were performed on these 948 genes within the physical interval of five stable unconditional QTL and five consistent QTL. Among the 778 genes with functional annotation information of homologous genes in *Arabidopsis*, to reduce candidate genes prediction errors, only genes whose homologous genes in *Arabidopsis* have been annotated to be involved in PH regulation, or genes that were previously proved to regulate PH, were included in the predicted candidate genes. Then, we finally predicted 14 candidate genes for cotton PH (Supplementary Table 10).

In this study, the gene *GH_D11G2212* was annotated as *AGAMOUS-like 29* in *Arabidopsis*, and the genes *GH_D11G2122* and *GH_D11G2351* were annotated as *AGAMOUS-like 104* in *Arabidopsis* (Supplementary Table 10). The homologous gene *Gh_D03G0922* of *Arabidopsis* was annotated as *AGAMOUS-like 8*, which had been proved to be related to cotton PH development (Su et al., 2018). The gene *GH_D11G2128* is annotated as gibberellin-regulated family protein in *Arabidopsis* (Supplementary Table 10). Gibberellin can regulate plant stem elongation and other developmental processes (Braun et al., 2019; Li and Li, 2019). The gene *GH_D09G1177* was annotated as auxin response factor 4 in *Arabidopsis*, and the gene *GH_D11G2121* was annotated as auxin-responsive *GH3* family protein in *Arabidopsis* (Supplementary Table 10). The gene *GH3* couples IAA to amino acids to maintain auxin homeostasis. It was indicated that the *GH3* gene participated in PH regulation and its high expression resulted in shorter plant (Yu et al., 2002; Staswick et al., 2005). The gene *GH_D11G2197* is annotated as squalene epoxidase 2 in *Arabidopsis* (Supplementary Table 10) and may be involved in sterol biosynthesis (Laranjeira et al., 2015). The genes *GH_D09G1074*, *GH_D11G2118*, *GH_D11G2301*, *GH_D11G2302*, *GH_D11G2303*, *GH_D11G2399*, and *GH_D11G2415* were annotated as cytochrome P450 in *Arabidopsis* (Supplementary Table 10). Cytochrome P450-related gene mutations may hinder the synthesis of brassinolide and cause plants to become shorter (Yang et al., 2014; Wu et al., 2016; Qi et al., 2017).

**FIGURE 5:**
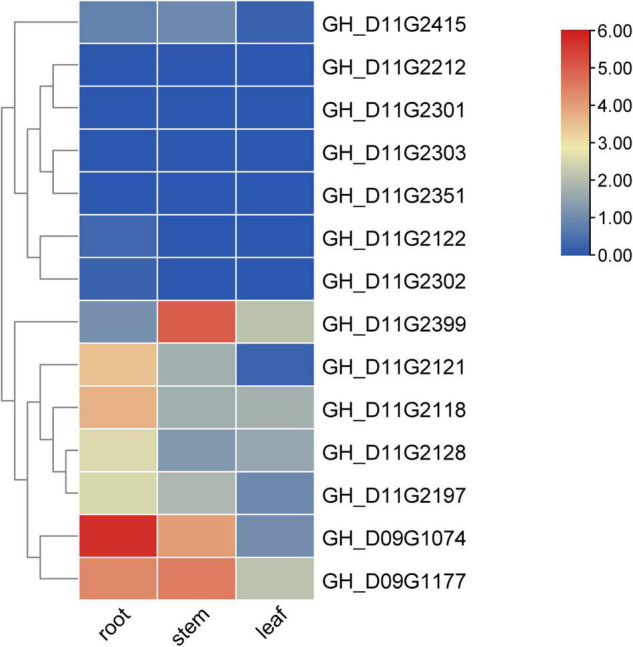
Expression information of candidate genes in TM-1. The value shown in the heatmap is log_2_(1+RPKM) based on RNA-seq data of TM-1. The data are the original expression data in the expression mode.

The gene expression heatmap indicated that the gene *GH_D09G1177* was specifically expressed in the stem and root of TM-1, the genes *GH_D11G2121*, *GH_D09G1074*, and *GH_D11G2118* were specifically expressed in the root of TM-1, and the gene *GH_D11G2399* was specifically expressed in the stem of TM-1 (Hu et al., 2019; Figure 5). Since the PH is mainly determined by the growth of the stem, we can give priority to the gene *GH_D11G2399*, which is preferentially expressed in the stem, and its expression level is significantly higher than that in other important tissues (leaf and root) of TM-1.

## Discussion

### Comparison With Previously Reported Plant Height Quantitative Trait Loci

Since quantitative traits are easily affected by the interaction between genes and the environments, the phenotypic data were usually collected from various environments to reduce the effect of environments. It can improve the accuracy of prediction by the phenotypic regression analysis on hundreds of thousands of mutations through a genome-wide regression model at the same time (de Los Campos et al., 2013). BLUP is a genome regression model method that is commonly used in animal and plant populations to improve prediction accuracy (de Los Campos et al., 2013; Huang et al., 2017). In this study, the BLUPs of 5 HZ environments, 4 DZ environments, and all environments were calculated for QTL mapping to improve the accuracy of QTL mapping. Among the 60 unconditional and conditional QTL detected in this study, 10 QTL were regarded as stable or consistent QTL. Furthermore, the annotation of homologous genes in *Arabidopsis* detected candidate genes with proved gene function involving PH regulation in these stable QTL confidence intervals. In addition, stable QTL detected at specific location was valuable for developing cultivars suitable to that location. The results of this study showed that 27 QTL were only detected in the HZ environment including a stable QTL (*UqPH-D02-1*) and a consistent QTL (*qPH-D13-1*), and 21 QTL were only detected in the DZ environment including a stable QTL (*UqPH-A09-1*).

Plant architecture is related to traits such as yield and fiber quality and has an important impact on high yield and good quality (Fu et al., 2019). Moreover, there are few QTL detected for PH in cotton, so it is necessary to use high-density genetic map to detect QTL for PH (Li et al., 2013; Liu et al., 2020). In this study, we used the constructed high-density genetic map and QTL mapping based on the PH phenotype data of the five generations of F_9_–F_13_, and a total of 60 PH QTL were mapped. To identify the newly discovered QTL in this study, the upland cotton PH QTL included in CottonGen^5^ (Huang et al., 2017; Liu et al., 2020; Yu et al., 2021), and PH QTL identified in recent years (Said et al., 2013; Huang, 2018; Su et al., 2018; Ma et al., 2019) were compared with the QTL of this work. The physical location of QTL is determined according to markers of confidence interval. If the physical intervals between QTL shared partially or fully overlapped, they are considered to be the same QTL. Due to the difference of marker types used in previous QTL mapping research, only QTL mapped with SNP markers were included in QTL comparison. It was found that 21 QTL (35.0%) mapped in this study were previously reported (Supplementary Table 6), including 12 unconditional QTL, eight conditional QTL, and one consistent QTL. The rest 39 QTL detected here were preliminarily considered as new cotton PH QTL. In addition, seven QTL were detected by BLUP, suggesting that using BLUP to detected QTL was reliable.

### Conditional Quantitative Trait Loci Mapping

The phenotypic changes of dynamic traits are characterized by the time, indicating that the genetic mechanism of these traits may also change over time (Wurschum et al., 2014). PH is a dynamic development trait, unconditional QTL is the cumulative effect from the initial time to t, conditional QTL can reveal the net expression effect of QTL from t-1 to t (Liu, 2011). In this study, QTL mapping was performed on nine environments and 3 BLUPs, with a total of 37 unconditional QTL and 28 conditional QTL mapped, and found that five QTL were both unconditional QTL and conditional QTL after the comparison. The number of QTL varies at different stages of PH development, most unconditional QTL were detected in the early and middle stages, while most conditional QTL were detected in the middle stages, indicating that PH QTL have spatiotemporal expression characteristics (Shang et al., 2015a,^2016^; Wang et al., 2019). The same QTL can also be expressed in different periods, for example, consistent and stable QTL *qPH-A08-1* was detected in 12 periods of t1, t2, t3, t4, and △t2-t3 of 5 environments, and *qPH-D09-1* was detected in five periods of t2, t3, t4, and △t1-t2 of 3 environments (Table 3). However, no QTL was found to be expressed in all stages of an environment, indicating that different QTL are expressed at different stages in the process of PH development.

## Conclusion

Plant height is a key plant architecture factor affecting cotton fiber yield and other traits. A high-density genetic map of 5,850 SNP markers was constructed using an upland cotton intraspecific recombinant inbred line (RIL) population. Based on phenotypic PH data from nine environments and three BLUPs, 37 unconditional QTL and 28 conditional QTL sharing five common QTL were identified for PH, including 21 previously detected PH QTL. Fourteen candidate genes for PH were predicted within 10 stable QTL intervals. This study shed substantial light on the genetic basis of cotton PH, will lay a foundation for further functional verification of candidate genes, and enable molecular selection toward ideal plant architecture.

## Data Availability Statement

The original contributions presented in the study are included in the article/Supplementary Material, further inquiries can be directed to the corresponding authors.

## Author Contributions

JW completed the phenotypic data collection, analyzed and summarized all the data, and wrote the manuscript. LM and JT participated in the phenotypic data collection. XW completed the candidate genes enrichment and wrote the results section. HZ, MX, YS, YZ, GZ, ZZ, YW, MC, and LW managed and collected the phenotype data. XLS and XZS designed the research. XLS revised the manuscript. All authors read and approved the final manuscript.

## Conflict of Interest

The authors declare that the research was conducted in the absence of any commercial or financial relationships that could be construed as a potential conflict of interest.

## Publisher’s Note

All claims expressed in this article are solely those of the authors and do not necessarily represent those of their affiliated organizations, or those of the publisher, the editors and the reviewers. Any product that may be evaluated in this article, or claim that may be made by its manufacturer, is not guaranteed or endorsed by the publisher.

## Footnotes

1
https://cottonfgd.org/

2
http://cotton.zju.edu.cn/

3
https://www.omicstudio.cn/tool

4
https://magic.novogene.com/customer/main#/login

5
https://www.cottongen.org/

## Supplementary Material

Supplementary Figure 1 The quantitative trait loci (QTL) allele effect for the *qPH-A08-1*, *qPH-A11-1*, *qPH-D07-1*, *qPH-D09-1* and *qPH-D13-1*related to PH. **(a)** Box plots for the *qPH-A08-1* related to PH. **(b)** Box plots for the *qPH-A11-1* related to PH. **(c)** Box plots for the *qPH-D07-1* related to PH. **(d)** Box plots for the *qPH-D09-1* related to PH. **(e)** Box plots for the *qPH-D13-1* related to PH.
