# Supplementary figures and images for: Dynamic Quantitative Trait Loci Mapping for Plant Height in Recombinant Inbred Line Population of Upland Cotton
Source: Front Plant Sci. 2022 Jun 9;13:914140. doi: 10.3389/fpls.2022.914140 (PMC9235862; doi:10.3389/fpls.2022.914140)

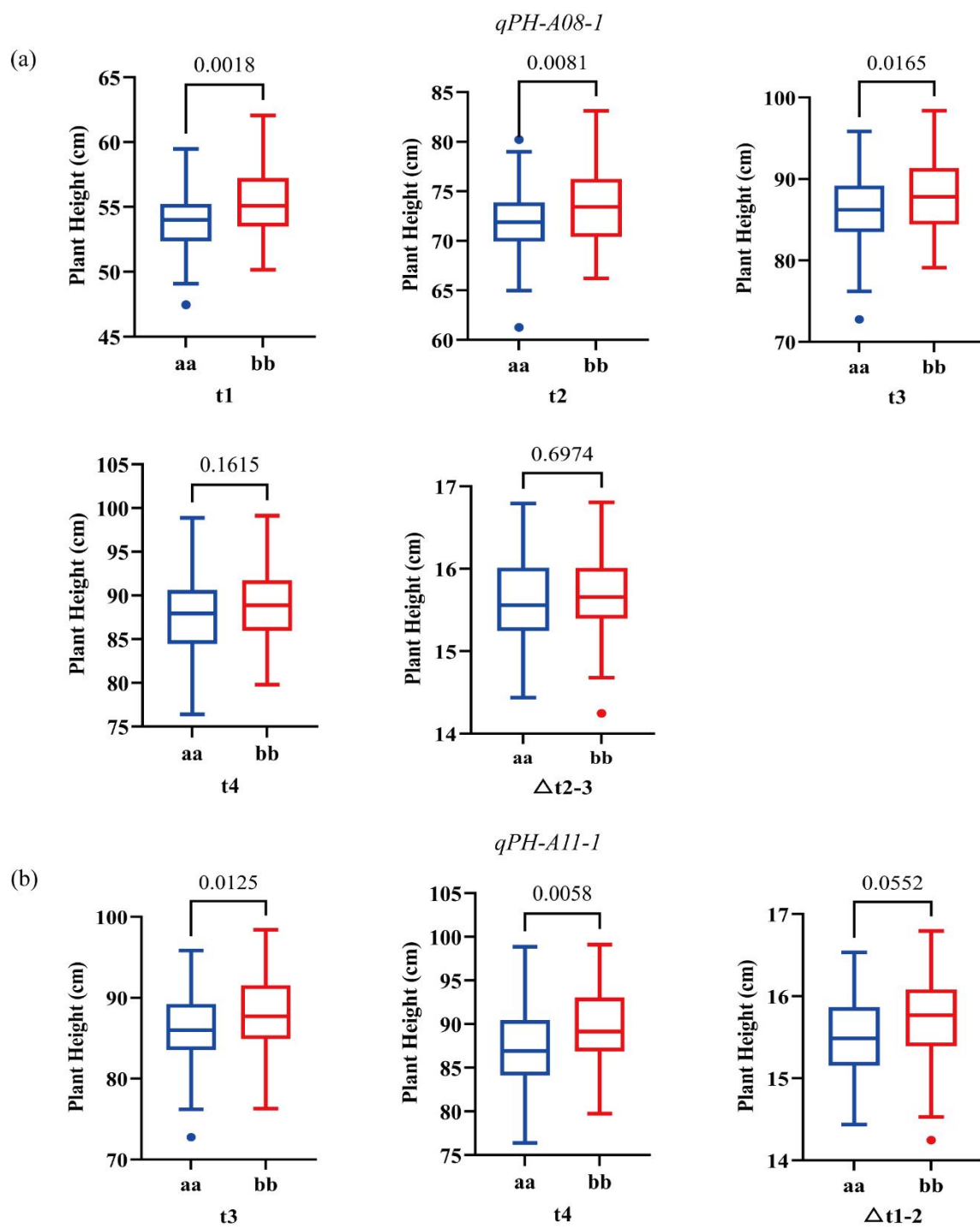

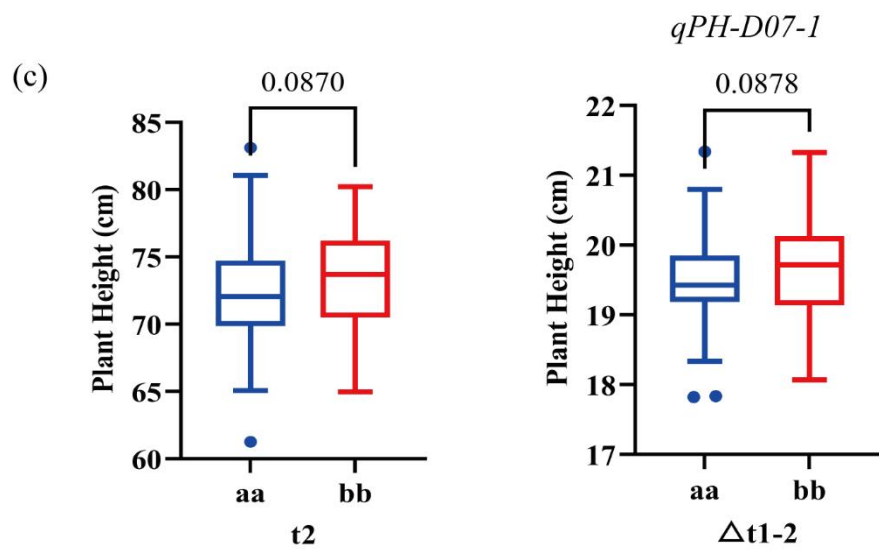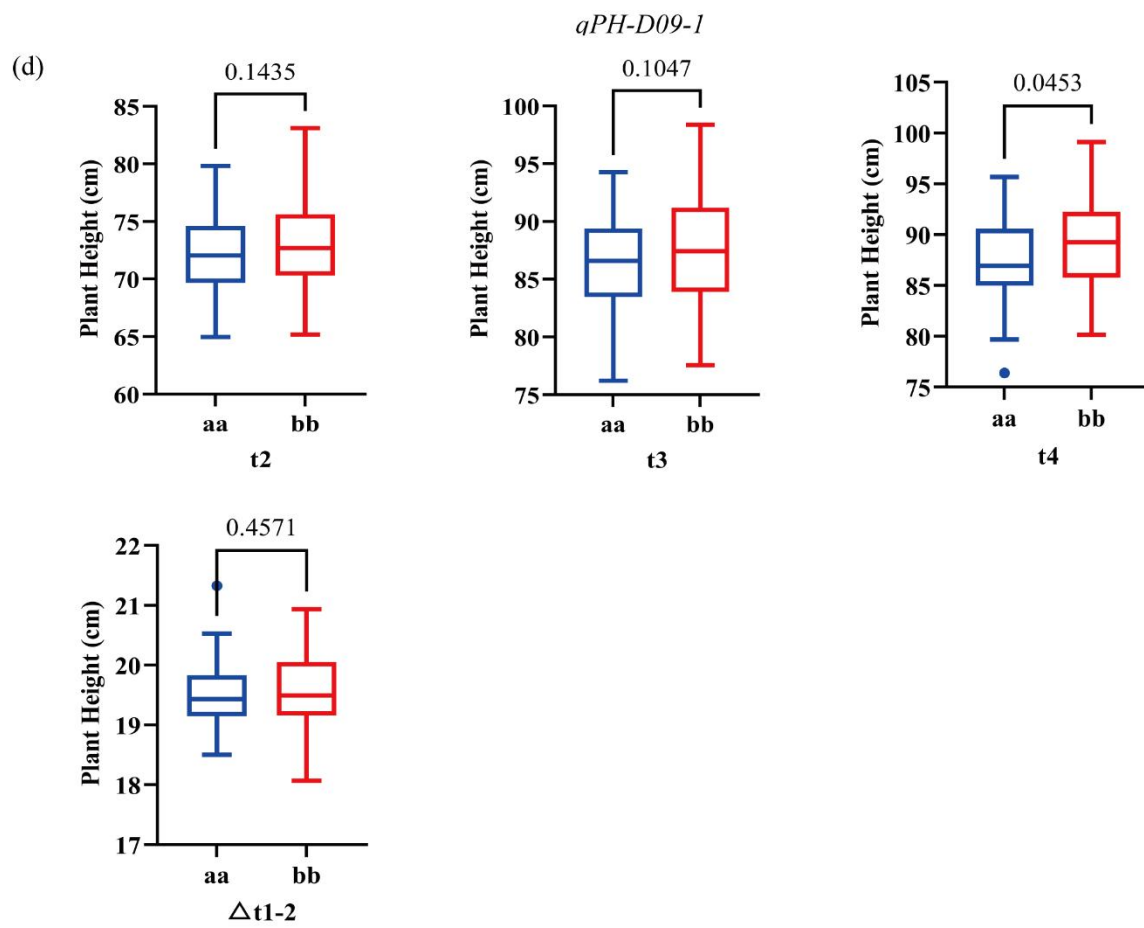

(e)

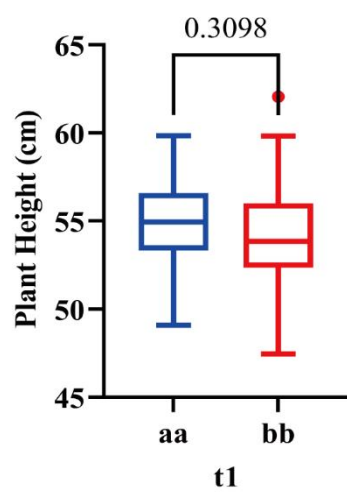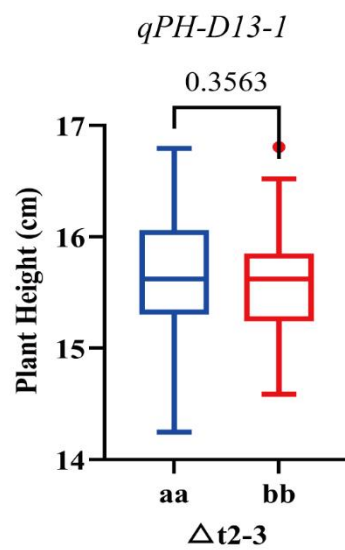

Supplement: Supplementary Figure 1 — The quantitative trait loci (QTL) allele effect for the qPH-A08-1, qPH-A11-1, qPH-D07-1, qPH-D09-1 and qPH-D13-1related to PH. (a) Box plots for the qPH-A08-1 related to PH. (b) Box plots for the qPH-A11-1 related to PH. (c) Box plots for the qPH-D07-1 related to PH. (d) Box plots for the qPH-D09-1 related to PH. (e) Box plots for the qPH-D13-1 related to PH. [file Image_1.pdf]
